# Modern therapies of nonsmall cell lung cancer

**DOI:** 10.1007/s13353-023-00786-4

**Published:** 2023-09-12

**Authors:** Andrzej Jachowski, Mikołaj Marcinkowski, Jakub Szydłowski, Oskar Grabarczyk, Zuzanna Nogaj, Łaz Marcin, Andrzej Pławski, Paweł Piotr Jagodziński, Bartosz Kazimierz Słowikowski

**Affiliations:** 1 Department of Biochemistry and Molecular Biology, Poznań University of Medical Sciences, Święcickiego 6 Street, 60-781 Poznań, Poland; 2 Institute of Human Genetics, Polish Academy of Sciences, Strzeszyńska 32 Street, 60-479 Poznań, Poland

**Keywords:** Lung cancer, NSCLC, Immunotherapy, Targeted therapy, CRISPR, Nanoparticles

## Abstract

Lung cancer (LC), particularly nonsmall cell lung cancer (NSCLC), is one of the most prevalent types of neoplasia worldwide, regardless of gender, with the highest mortality rates in oncology. Over the years, treatment for NSCLC has evolved from conventional surgery, chemotherapy, and radiotherapy to more tailored and minimally invasive approaches. The use of personalised therapies has increased the expected efficacy of treatment while simultaneously reducing the frequency of severe adverse effects (AEs). In this review, we discuss established modern approaches, including immunotherapy and targeted therapy, as well as experimental molecular methods like clustered regularly interspaced short palindromic repeat (CRISPR) and nanoparticles. These emerging methods offer promising outcomes and shorten the recovery time for various patients. Recent advances in the diagnostic field, including imaging and genetic profiling, have enabled the implementation of these methods. The versatility of these modern therapies allows for multiple treatment options, such as single-agent use, combination with existing conventional treatments, or incorporation into new regimens. As a result, patients can survive even in the advanced stages of NSCLC, leading to increased survival indicators such as overall survival (OS) and progression-free survival (PFS).

## Introduction

According to the latest data from GLOBOCAN, in 2020, more than 2.2 million people were diagnosed with lung cancer (LC), and almost 1.8 million deaths were noted worldwide. It made LC the most common cause of cancer-related death globally (Sung et al. 2021). Even with the increased smoking cessation and subsequent decrease in cases among men in developed countries, the overall statistics have grown since the last analysis in 2018 (Bray et al. 2018). National Cancer Institute’s Surveillance, Epidemiology, and End Results (SEER) program indicates that the 5-year survival rate in the USA (2010–2016) was 20.5%, which is a significant improvement compared to the rate from 1975, which was 11.5% (Thandra et al. 2021). This phenomenon can be attributed to the rapid development of diagnosis and treatment methods such as computed tomography (CT) and the introduction of immunotherapy and targeted therapies for LC. There are several risk factors responsible for LC development, such as age, gender, race/ethnicity, family history, tobacco smoking, cannabis smoking, environmental pollution, infections, inflammation, and chronic obstructive pulmonary disease (Thandra et al. 2021).

Tobacco smoking is the most critical risk factor in LC formation, as it accounts for over two-thirds of deaths and is a traceable cause in up to 80% of cases (Sung et al. 2021; Thandra et al. 2021). Combustion products contain various carcinogenic substances, such as polycyclic aromatic hydrocarbons and tobacco-specific N-nitrosamines. The presence of these carcinogens may lead to the activation of specific oncogenes (e.g. *KRAS*) and inhibition of tumour suppressor genes (TSGs) (Dela Cruz et al. 2011; Akopyan and Bonavida 2006).

LC is divided into types and subtypes based on the histological features of the tumour cells. Small cell lung carcinoma (SCLC) and nonsmall cell lung carcinoma (NSCLC) are the two main types of LC, with NSCLC being much more common (85% of all LC cases) (Veronesi et al. 2017). NSCLC’s further subtypes include adenocarcinoma (LUAD), squamous cell carcinoma, and large cell carcinoma. In 2015, WHO introduced another subtype, neuroendocrine tumours, which consist of SCLC, large cell neuroendocrine carcinoma, and carcinoid. LUAD and squamous cell carcinoma are the most common types of lung tumours. Current diagnosis is more often based on immunochemistry than morphology, with biomarkers being the determining factor in situations of uncertainty (Veronesi et al. 2017). Genetic screening is becoming more common in LC with the development and cost reduction of next-generation sequencing (NGS). Specific mutations and gene alterations have been found to influence lung tumorigenesis. Those alterations are present in genes which encode proteins such as the epidermal growth factor receptor (EGFR), Kirsten rat sarcoma virus (KRAS), anaplastic lymphoma kinase (ALK), B-RAF, and ROS1.

Recognition of those mutations has facilitated the development of targeted therapy. Most defects affect signalling pathways within the cell due to tyrosine kinase (TK) activation. Monoclonal antibodies (mAbs) and tyrosine kinase inhibitors (TKIs) are used in selected patients to restrain the effects of mentioned alterations (Larsen et al. 2011). Epigenetic modifications have also been found to affect LC development. Hypermethylation of certain TSGs, due to increased levels of DNA methyltransferases, prevents them from being expressed and impacts lung tumorigenesis from its early stages (Langevin et al. 2015). There is also evidence of hypomethylation in the CpG sequences, which may lead to increased recombination during proliferation and chromosome instability. Some chromatin structural changes can be reversed, hence the potential for chromatin modifiers to be used in targeted therapy (Mehta et al. 2015). The main aim of this brief review is to collect and introduce the potential option of available, modern ways of battles with LC.

### Conventional therapies

Due to the high mortality rate of NSCLC, there is a great need to develop new, more effective ways of curing patients (Siegel et al. 2014). New therapies begin to play a more significant role in NSCLC treatment. Nonetheless, classic approaches such as surgery, radiotherapy, and chemotherapy are still commonly used worldwide. Ongoing research on traditional therapies allows improving and using them more effectively. In this section, we want to outline the most common conventional treatment approaches briefly.

### Surgery

Surgical removal of a tumour has been the golden standard for patients in the early stage of NSCLC, but only if the tumour is resectable and the patient can survive the surgery (Zappa and Mousa 2016). The operation consists of removing the lobe, segment, or entire lung, depending on the tumour size, location, and level of the pulmonary reserve (Tandberg et al. 2018).

The oldest surgical intervention technique is open, posterolateral thoracotomy (conventional), and muscle-sparing thoracotomy (Wang et al. 2019). However, due to technological development, video-assisted thoracic surgery (VATS) has gained popularity. According to numerous studies, VATS appears significantly superior to thoracotomy, with fewer removed lymph nodes, less intraoperative bleeding and trauma, fewer postoperative complications, shorter hospitalisation, faster rehabilitation, and better 3- and 5-year survivals (Wang et al. 2019; Dziedzic et al. 2021). Usability limitations of VATS may arise from low experience in using this technique and higher costs. Nonetheless, available data shows that shorter hospitalisation and reduced postoperative healthcare requirements lower the costs of using VATS (Wang et al. 2019). In the past two decades, three-port VATS has been positively established and has become a conventional treatment for NSCLC. This relatively new variant of VATS involves making one 4-cm-long incision. It is a less invasive form of lung surgery proven to reduce trauma, decrease postoperative pain, and shorten the time needed for rehabilitation. However, it requires the extensive experience of the surgeon (Wang et al. 2017).

The latest and most challenging technique of surgical tumour removal in NSCLC is robot-assisted thoracic surgery (RATS). The biggest problems encountered in using RATS are related to the costs of this procedure and learning how to perform this technique (Lee et al. 2021). Robotic lobectomy may be recommended for operations requiring greater precision because robotic arms can provide more precise movements. The only systems currently available for RATS are da Vinci Systems (Veronesi 2015). According to most studies that compare RATS and VATS, there are no differences in mortality, overall survival (OS), disease-free survival, and intraoperative and postoperative parameters (Ma et al. 2021; Guo et al. 2019). However, a meta-analysis published in December 2020 suggests that RATS may result in better OS (Wu et al. 2021). This research implies that RATS is a safe and practicable technique in NSCLC treatment.

### Radiotherapy

Radiotherapy is another important form of NSCLC treatment. It can be applied in every stage of NSCLC as adjuvant or neoadjuvant therapy (Uzel et al. 2019). Technological development and diagnostic imaging have made radiotherapy more precise and safer for patients. Research shows the superiority of three-dimensional conformal radiotherapy over two-dimensional therapy, which is characterised by poor control of tumour location (Chang et al. 2008). One of the latest techniques of this treatment is image-guided radiotherapy (IGRT). It can be used with four-dimensional CT or positron emission tomography, allowing tumour imaging before and during the procedure. IGRT maximised the precision of the radiation beam and reduced damage to healthy near-tumoural tissue (Kilburn 2016). One factor that can limit radiotherapy’s precision is respiratory movement during irradiation. Respiratory-gated conformal radiotherapy (RGRT) can be used to limit the impact of this factor. This technique involves sending the radiation beam toward the tumour during a predefined gate or blocking the patient’s breathing. Studies have shown that RGRT can reduce acute and late toxicity during chest irradiation. Nevertheless, this therapy is associated with higher financial costs (Giraud et al. 2011).

There are several techniques for radiotherapy: conventional with low doses of radiation and prolonged treatment time, stereotactic body radiation therapy (SBRT) with high doses but short treatment time, and intensity-modulated radiation therapy with modulated doses and longer treatment time. SBRT is a more complex technique, but many studies have shown that it is more convenient for patients due to shorter treatment times and lower morbidity, especially when used against peripheral tumours. Cancer foci located centrally are more challenging because of the increased risk of damage to the mediastinal structure (Tandberg et al. 2018; Donovan and Swaminath 2018; Kepka and Socha 2021).

Radiotherapy can be performed alone or with other types of NSCLC treatment, such as surgery, chemotherapy, immunotherapy, therapy with nanoparticles, targeted therapy, and CRISPR-Cas9 therapy.

### Chemotherapy

Chemotherapy is commonly used in the IV stadium of NSCLC. Drugs used in chemotherapy, including LC, utilise the neoplastic cells’ capability of more intense mitosis compared to host cells, which makes these medicaments more toxic to the tumour. Platinum-based drugs are frequently used as they preferentially adduct with the N7 atom on guanine and adenosine. This bonding process impedes DNA replication and transcription, ultimately leading to apoptosis. Research has shown that the most effective outcomes are achieved through 4 rounds of chemotherapy that include platinum (Masters et al. 2015; Gridelli et al. 2004; Gerber and Schiller 2013).

The type of drugs administered to patients depends on their performance status (PS, scale 0–5), which describes their ability to function independently. According to The American Society of Clinical Oncology, the best combination of cytotoxic medicaments for patients with PS of 0 or 1 is *cisplatin* or *carboplatin* with *paclitaxel*, *gemcitabine*, *docetaxel*, *vinorelbine*, *irinotecan*, or *pemetrexed* (Masters et al. 2015). Patients treated this way can survive 8–10 months (Zappa and Mousa 2016). Nonetheless, a combination of these medicaments should be determined individually. Patients with a PS of 2 may be effectively treated only with nonplatinum drugs (Gridelli et al. 2004). Patients with a PS of 3 usually do not benefit from chemotherapy due to adverse effects (AE) and worse quality of life (Zappa and Mousa 2016).

Four of the most discussed forms of chemotherapy treatment are standard, sequential, alternating, and maintenance chemotherapy. All of them are effective in different groups of patients. However, it should be noted that only standard therapy is a validated and non-experimental method of chemotherapy (Grossi et al. 2007).

Sequential chemotherapy is based on applying non-cross-resistant medicaments in a specific sequence. It allows the application of more drugs with reduced toxicity by optimisation of dose of each medicament (Grossi et al. 2007). According to studies, sequential chemotherapy with a single agent followed by another can be promising for elderly feeble patients who do not qualify for platinum-based chemotherapy. For patients with good PS, an optimistic sequence of medicaments is a platinum-based doublet followed by a single, third-generation agent. The preliminary results of studies on this method have shown lower toxicity in this method with no differences in progression-free survival (PFS) and OS between this method and standard chemotherapy (Castro et al. 2002; Clark et al. 2001; Hosoe et al. 2003; Binder et al. 2007; Grossi et al. 2004; Edelman et al. 2004; Rinaldi et al. 2005; Chiappori et al. 2005).

Alternating chemotherapy is based on applying non-cross-resistant medicaments or their combination for a predetermined number of cycles or until progression. Optimal doses should be used as early as possible. Alternating administration of medicaments can reduce the toxicity of specific drugs (Grossi et al. 2007). Nonetheless, studies have shown that alternating chemotherapy is less effective for patients with good PS than standard therapy (Grossi et al. 2007). Despite this, several phase II trials have shown that alternating single-agent chemotherapy can be promising for elderly patients with bad PS (Mattson et al. 2000; Aguiar et al. 2005; Gridelli et al. 2007).

Maintenance chemotherapy is based on the administration of additional medicaments after planned in-advance chemotherapy. The drug used in this method can be one of those used in the standard cycle or a non-cross-resistant drug. The most effective outcome of maintenance chemotherapy is observed when the drug has been efficient during previous induction chemotherapy (Grossi et al. 2007). Studies have shown that maintenance chemotherapy can increase PFS, but OS is often not improved (Gerber and Schiller 2013). Two of the most promising drugs used in maintenance treatment are *pemetrexed* and *bevacizumab*. They present better OS; however, further research is required in this field (Gerber and Schiller 2013).

Chemotherapy can be combined with radiotherapy, immunotherapy, targeted therapy, surgery, and nanoparticle therapy. Currently, the most extensive research on cancer treatment has been on the combination of chemotherapy and surgical methods. The studies demonstrate that neo-adjuvant chemotherapy is primarily administered for the early stages of lung cancer. The procedure enables the reduction of tumour mass, making the operation feasible. Research shows that adjuvant chemotherapy is highly successful in decreasing mortality rates among patients diagnosed with stage IB cancer. There is a possibility that adjuvant chemotherapy, utilising platinum compounds, could become the standard for stage IB–IIIA of NSCL treatment.

### Immunotherapy

Immunotherapy is one of the latest forms of cancer treatment and focuses on the patient’s immune system. Neoplastic cells have developed numerous ways to avoid apoptosis and destruction by the immune system, so the immune system needs a specific boost provided by immunological treatment. There are several branches of immunotherapy, and the two focus on CD8 + T cells and factors that modify their function, i.e. cytokines—small proteins responsible for interactions between immune system cells. Interleukin-2 is the most effective cytokine regarding T cell activation, which can eventually lead to a reduction of tumour size (Rosenberg et al. 1984, 1985; Rosenberg 2014; Taniguchi et al. 1983). Another option is using immune checkpoint inhibitors (ICIs). Their mechanism of action includes binding with receptor proteins located on the cytoplasmatic membrane of CD8 + T cells. These receptors enable cancer cells to avoid an immune system response by binding with cancer-produced proteins. Immunotherapeutic mAbs prevent from binding with these particles, increasing the capability of T cells to attack the tumour. The most commonly used antibodies in LC treatment are *ipilimumab*, *pembrolizumab*, and *nivolumab* (Steven et al. 2016). However, the tumour microenvironment may have highly immunosuppressive effects, which is the main obstacle in applying such medicaments. This area of research is being extensively investigated; learning about and eliminating these effects could be a promising possibility in helping the immune system destroy neoplastic cells (Steven et al. 2016).

Another successful immunotherapy method is adoptive cell therapy, based on genetic modification and proliferation of a patient’s T cells so they can effectively attack neoplastic cells. After modification, T cells are moved back to the patient. This approach has proven effective, but several potentially dangerous AEs currently limit the use of adoptive cell therapy (Dobosz and Dzieciątkowski 2019). Other promising immunotherapy options are oncolytic viruses and vaccines. The first one can be used to boost the immune system. Genetically modified viruses, which can penetrate only neoplastic cells, can cause their destruction. Molecules released during the disintegration of cancer cells stimulate the immune system to respond (Oiseth and Aziz 2017). Years ago, much hope arose among the scientific community that vaccines could also be used in preventing and treating cancers. Currently, there are only a few available and approved anticancer vaccines, but many of them, both allogeneic and autologous, are being investigated, and high hopes are placed on them (Dobosz and Dzieciątkowski 2019).

Immunotherapy appears to be extremely promising. Thanks to a large body of research conducted worldwide, it will be increasingly developed and may become a primary form of cancer treatment.

### Immunotherapy in lung cancer

Immunotherapy and its incorporation of ICIs is the most promising branch of LC treatment. There have been attempts at developing alternative immunotherapeutic drugs, but none of them reached the efficacy achieved by ICIs (Steven et al. 2016).

### Efficacy and safety

Compared to the standard NSCLC chemotherapy treatment approach, immunotherapy has proven to be more effective. Generally, ICIs may contribute to better OS and PFS values in previously treated NSCLC patients and be invaluable as first-line treatment (Reck et al. 2021; Zhou et al. 2021; Wang et al. 2020a). In the clinical study of the metastatic LC, the use of ICIs alone with programmed death ligand-1 (PD-L1) expression ≥ 50% versus chemotherapy resulted in a doubling of the median OS and 5-year OS rate (Reck et al. 2021). Also, when combined with the cycles of standard chemotherapy, results have shown an increased OS and PFS (Reck et al. 2021). Additionally, clinical trials showed the overall relative safety of this approach compared to the conventional approach, even in cases of combining immunotherapies. However, this depends on the individual patient’s characteristics and draws particular attention to potential AEs (hui Jia et al. 2020; Zhao et al. 2021). To summarise the abovementioned results, many approaches combine treatment methods to achieve the best possible efficacy (Huang et al. 2021). Nevertheless, it is significant to consider the sex of a patient, as the analysis of clinical trials in cases of NSCLC and melanoma denotes that the particular ICI efficiency (i.e. *pembrolizumab)* decreases for women (Conforti et al. 2018).

Observed possible mild AEs include nausea, fatigue, dyspnea, and decreased appetite; however, given the antibody nature of immunotherapeutic drugs, AEs can escalate to severe conditions such as pneumonitis, hepatitis, colitis, psoriasis, transient thyroid malfunction, and diabetes mellitus (Zarogoulidis et al. 2017; Godwin et al. 2017; Iwama and Arima 2017). Furthermore, this implies that careful observation must continue long after termination of the treatment because of the hazard of developing long-term autoimmune conditions (Suresh et al. 2018; Puzanov et al. 2017).

### The mechanism

The immune system destroys cancer cells through tumour-specific T cells, utilising the tumour-specific differentiation 8 T (CD8 T) cluster to distinguish hostile cells from the host cells. This response requires the presentation of tumour antigens by the antigen-presenting cells (Steven et al. 2016). In LC, several steps of this system may be disturbed, impairing the immune response, therefore allowing cancer cells to thrive. It may include an insufficient antigen expression on the surface of the tumour cells, a disturbance in the antigen-presenting process or local immunosuppressive conditions. To further suppress the immune response, cancer cells may exploit the ICI system by expressing specific ligands on their surface, such as the PD-L1 or B7 compound family, which bind to corresponding receptors on the T cells and inhibit their anti-tumour activity (Steven et al. 2016; Dobosz and Dzieciątkowski 2019; Oiseth and Aziz 2017).

Additionally, regulatory T cells may favour immunosuppression in the presence of specific cancer molecules. Furthermore, prolonged exposure to increased concentrations of hostile antigens or inflammatory signals (as in chronic infections or cancers) may lead to T cell exhaustion, making T cells lose their effector functions and express inhibitory signals (Wherry and Kurachi 2015). Both ICIs and T cell exhaustion can be reversed using immunotherapeutic drugs, allowing the reinduction of the immune cell response.

### PD-1/PD-L1 axis

**Fig. 1:**
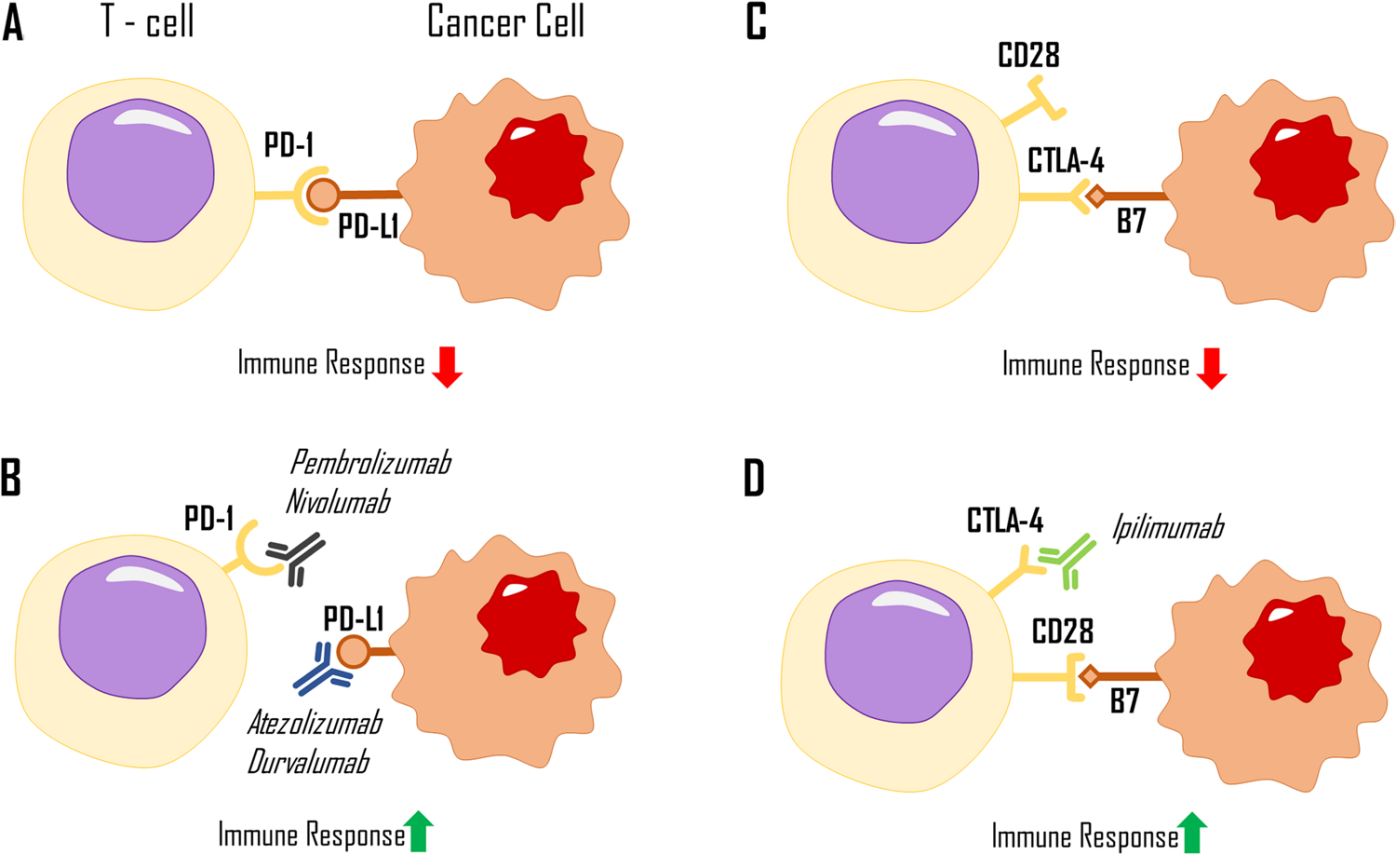
Interaction between immune checkpoint inhibitor ligands, their corresponding receptors (**A**, **C**), and most commonly used immunotherapeutic drugs (**B**, **D**). PD-1, programmed death receptor; PD-L1, programmed death ligand 1; CTLA4, cytotoxic T-lymphocyte associated protein 4; B7, B7 compound family molecules; CD28, cluster differentiation protein 28; immunoterapeutic drugs are portrayed as symbols of antibodies, visualising their immune checkpoint blocking capabilities

The most crucial immunological checkpoint exploit in LC involves the activation of a T cell programmed death receptor-1 (PD-1). PD-1 can be expressed in many haematopoietic and epithelial cells and the tumour microenvironment. Matching PD-L1 binds to the PD-1 receptor located on the surface of the T cell, downregulating its immune response (Fig. 1A). In NSCLC, a matching PD-L1 may be expressed in approximately half of the cancer cells, making the tumour highly susceptible to immunotherapy (Steven et al. 2016). On the other hand, programmed death ligand-2 (PD-L2) is located on Th2 cells, helping to mediate the inflammation.

Depending on the specific drug, immunotherapeutic drugs in the form of mAbs adhere to either PD-L1 or its receptor. This binding prevents the immunosuppressive T cell signal by rendering them unable to bind together (Xia et al. 2019). The leading pharmaceuticals that use this axis are IgG-type antibodies such as *atezolizumab* and *durvalumab*, which target PD-L1, and *nivolumab* and *pembrolizumab*, which target the PD-1 receptor (Fig. 1B). These drugs differ in their half-life, accompanying adverse effects, and possible immune-mediated adverse effects.

A crucial criterion of immunotherapy is the degree of PD-L1 expression on the surface of cancer cells, where PD-L1 ≥ 50% is the most favourable (Nasser et al. 2020). Individual predispositions, such as specific biomarkers and genetic landscapes, should be evaluated to ensure the best treatment option for a given patient (Rizvi et al. 2015). In the case of *pembrolizumab*, diagnostic trials include assessing tumour mutational burden, PD-L1 expression, and T cell response gene expression profile (Cristescu et al. 2018). Analysing these variables allows tailored therapy to a specific patient, advancing it into personalised precision therapy territory. The best-case scenario assumes treatment options with minimal use of chemotherapy or chemotherapy-free. However, knowing what is beneficial to a patient is most relevant, hence the importance of individual testing. Clinical trials, such as KEYNOTE (*pembrolizumab*), CHECKMATE (*nivolumab* + *ipilimumab*), and Impower (*atezolizumab*), confirm the efficacy of ICIs both as a monotherapy and in combination with standard chemotherapy such as *cisplatin*/*carboplatin* or *paclitaxel*, resulting in an overall increased overall survival median reaching 30 months. This makes it one of the most successful and currently best in use option for first-line treatments in NSCLC (Nasser et al. 2020).

### CTLA-4 axis

Another important immune checkpoint involves the cytotoxic T-lymphocyte-associated protein-4 (CTLA-4) axis, which is one of the earliest and most studied ICIs. This receptor is located on the surface of T regulatory cells alongside CD28. These two compete for adherence to B7 compound family molecules, such as CD80 and CD86 (Fig. 1C). The CTLA-4 receptor has a higher affinity for these ligands than CD28 and favours the downregulation of the immune response. *Ipilimumab*, a fully human kappa mAb used in treating melanoma, renal, and hepatic carcinoma, binds to the CTLA-4 receptor, making it unable to attach to its ligand, resulting in a more robust immune response due to the lack of prior-established inhibition (Fig. 1D) (Nasser et al. 2020). This drug can be used in combination with PD-1/PD-L1 checkpoint inhibitors and standard chemotherapy while still producing beneficial results. In a clinical trial of CheckMate 9LA, after a 2-year update, treatment with *nivolumab* (PD-1), *ipilimumab* (CTLA-4), and chemotherapy resulted in higher overall survival (OS) and double the progression-free survival (PFS) compared to standard chemotherapy (Reck et al. 2021). Case studies have also observed a correlation between radiotherapy and CTLA-4 blockade immunotherapy, showing increased efficacy when both are combined in treatment (Formenti et al. 2018).

### Other immunotherapies

There have been attempts at stimulating the immune system through Toll-like receptors located on the surface of B cells and plasmacytoid dendritic cells (Steven et al. 2016).

### VEGF-targeted therapy

In NSCLC, cancer cells produce vascular endothelial growth factor (VEGF), particularly two forms of this molecule, VEGF-A and VEGF-B, which leads to intense angiogenesis and a better blood supply for tumour cells, enabling their growth and proliferation (Bonnesen et al. 2009; Holm et al. 1996). Targeting VEGF and its receptors can suppress angiogenesis and slow tumour progression by reducing the oxygen and nutrient supply to the tumour (Yamazaki and Morita 2006). VEGF is a glycoprotein produced by various cells, including macrophages, keratinocytes, endothelial cells, mesangial cells, platelets, and tumour cells (Sunderkotter et al. 1994; Rosen 2002; Duffy et al. 2013; Zhang et al. 2022). There are several forms of VEGF, including VEGF-A, VEGF-B, VEGF-C, VEGF-D, VEGF-E, and VEGF-F (Lohela et al. 2009). Hypoxia-induced factor (HIF) is a crucial factor for VEGF secretion, which is produced in response to oxygen deprivation. VEGF binds to the vascular endothelial growth factor receptor (VEGFR) to initiate the pathway. VEGFR is a tyrosine kinase receptor with three domains: intracellular (with TK activity), transmembrane, and extracellular (Ferrara et al. 2003). The binding of VEGF to the extracellular domain activates the intracellular domain, leading to the phosphorylation of signalling proteins essential for cell migration, proliferation, and survival (Kowanetz and Ferrara 2006; Neufeld et al. 1999).

VEGFR1 and VEGFR2 are the two most important receptors in the VEGFR group, expressed on the surface of various cells, including endothelial cells (Yamazaki and Morita 2006; Neufeld et al. 1999). Both receptors bind VEGF, but VEGFR1 shows a ten times higher affinity for VEGF than VEGFR2. However, VEGFR2 has stronger TK activity, and some studies suggest that VEGFR1 regulates VEGFR2 function (Ferrara 2004; Cébe-Suarez et al. 2006). There is also VEGFR3, which plays a role in lymphangiogenesis and is located on the surface of endothelial cells in lymphatic vessels (Hamrah et al. 2004).

**Fig. 2:**
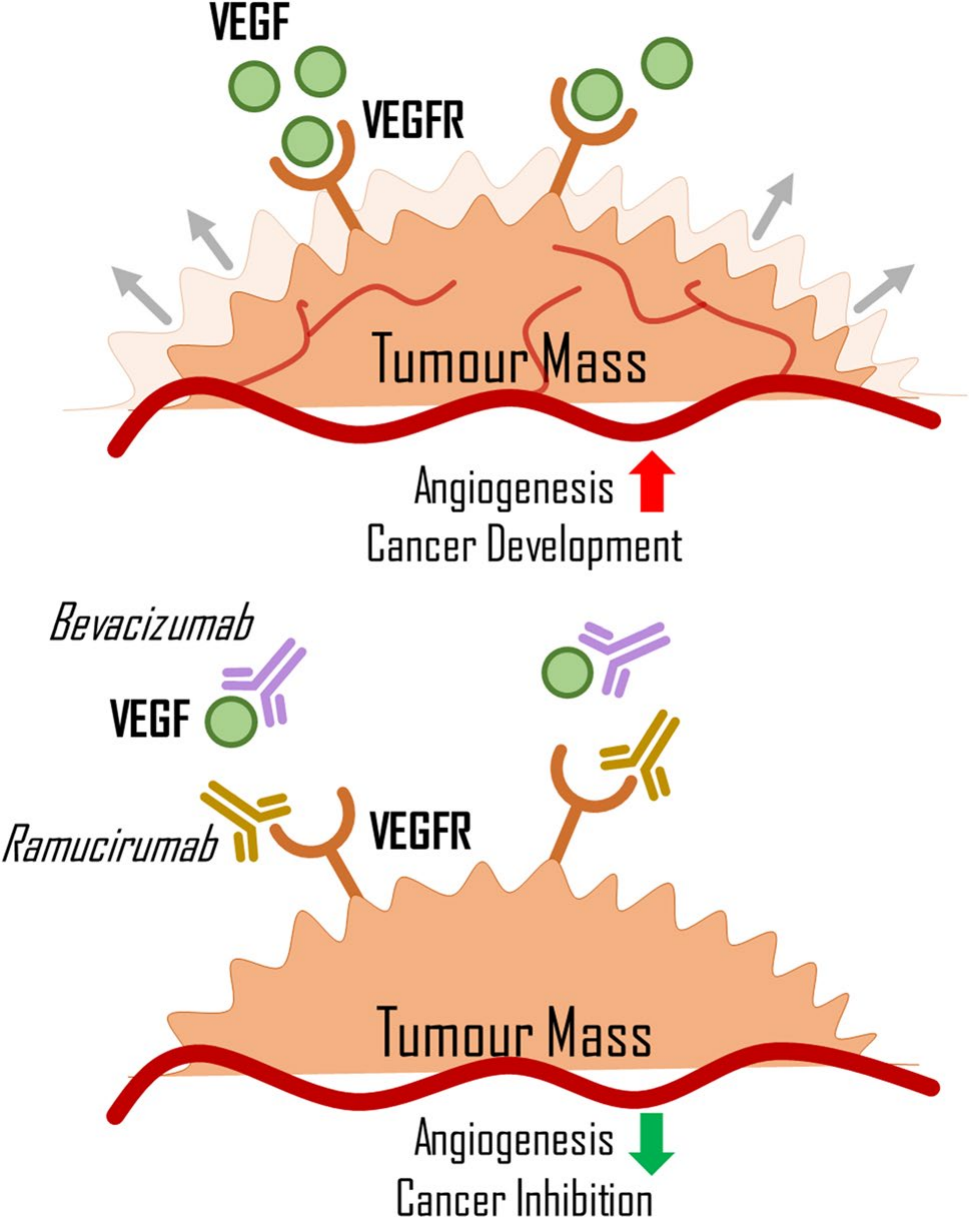
Interaction of VEGF and VEGFR with immunotherapeutic drugs. VEGF, vascular endothelial growth factor; VEGFR, vascular endothelial growth factor receptor; immunoterapeutic drugs are portrayed as symbols of antibodies, visualising their blocking capabilities. Red lines represent blood vessels; grey arrows visualize expanding tumour mass

During angiogenesis, VEGF initially induces vasodilation and increased vessel permeability. Next, endothelial cells proliferate and migrate to a site where a new capillary will be formed (Carmeliet 2000). Blood then flows through the preexisting vessel to the new vessel. Angiogenesis is a crucial process, not only important in wound healing or embryogenesis but also in carcinogenesis. Tumour cells require oxygen and nutrients to grow and proliferate, and new capillaries deliver these compounds. However, angiogenesis in tumours differs from that in healthy tissues. New vessels grow incoherently, often spiralling, and their permeability increases (Dudley 2012; Carmeliet 2005). This can lead to a weaker oxygen supply to the tumour cells, resulting in hypoxia. As a result, cells will produce more HIF, intensifying VEGF secretion and angiogenesis (Fig. 2) (Carmeliet 2005). Some monoclonal antibodies (mAbs), such as *bevacizumab* and *ramucirumab*, which can be used in LC treatment, operate by blocking angiogenesis.

*Bevacizumab* is a humanised monoclonal antibody that targets the VEGF-A molecule, preventing its attachment to its receptor and potentially inhibiting angiogenesis and tumour growth (Li et al. 2014; Garcia 2020). It is often used in combination with drugs like *erlotinib* or another monoclonal antibody, such as *atezolizumab*, in the treatment of NSCLC (Garcia 2020; Socinski et al. 2018; Cohen et al. 2005). While research shows that combination therapy with *bevacizumab* is generally safe, it can negatively affect the body, such as hypertension or pulmonary haemorrhage (Cohen et al. 2005; Piperdi et al. 2014). This is because *bevacizumab* reduces the level of VEGF, which impairs the remedial ability of the endothelium and weakens blood vessels, increasing the risk of rupture (Kamba and McDonald 2007; Johnson et al. 2004). *Ramucirumab* is another monoclonal antibody that has antiangiogenic properties. It binds to the extracellular domain of VEGFR2, preventing VEGF from binding to its receptor and blocking the formation of new blood vessels (Fig. 2) (Garon et al. 2014; Skobe et al. 1997). Recently approved in May 2020 for LC therapy, *ramucirumab* can be used with *erlotinib* when an activating mutation of EGFR is present in cancer cells (Nakagawa et al. 2019). As a newer drug in LC therapy, there is still some risk associated with its use*.*

### Pathway targeted therapy

**Fig. 3:**
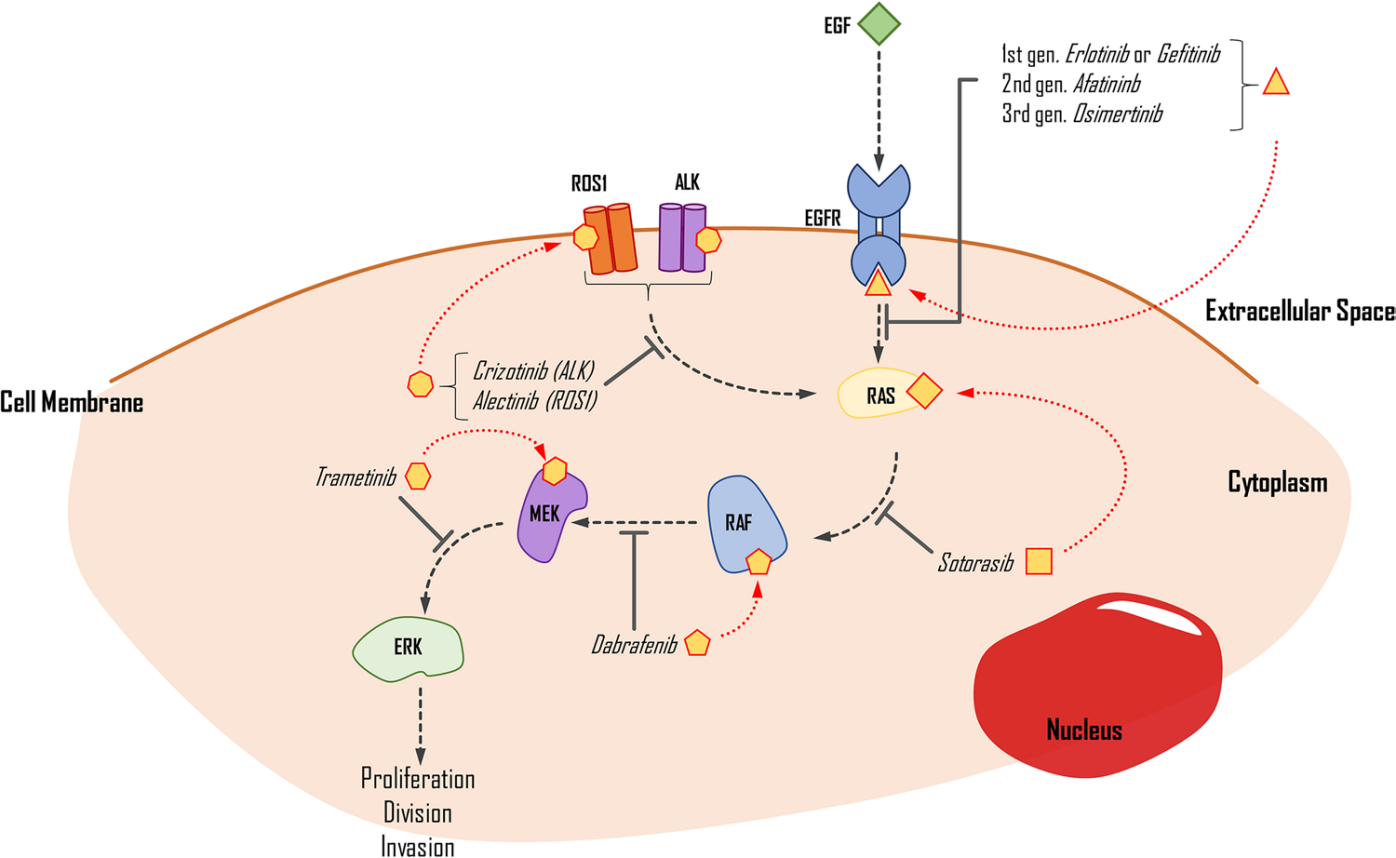
The simplified impact of selected drugs on the inhibition of the EGFR pathway. Medication is applied when proper mutations are detected in the genes that encode the pathway proteins. EGF, epidermal growth factor; EGFR, epidermal growth factor receptor; ROS1, proto-oncogene C-Ros 1; ALK, anaplastic lymphoma kinase; RAS, KRAS proto-oncogene; RAF, B-Raf proto-oncogene; MEK, mitogen-activated protein kinase kinase 1; ERK, mitogen-activated protein kinase kinase 3

Targeted therapy for NSCLC focuses on identifying mutations in the EGFR and MAPK/ERK pathways, along with ALK and ROS1 gene fusions (Fig. 3). These mutations cause excessive stimulation of cell proliferation, differentiation, and growth, ultimately leading to carcinogenesis. Multiple studies demonstrate that these mutations can exist separately or concurrently. Molecular testing has confirmed that most forms of NSCLC, particularly LUADs, are positively correlated with mutations or alterations in *EGFR*, *KRAS*, *ALK*, *ROS1*, and *B-RAF*, in decreasing frequency. Polymerase chain reaction and NGS, coupled with fluorescence in situ hybridisation (FISH), are widely employed for diagnosing these mutations in NSCLC patients (Dugay et al. 2017; Dogan et al. 2012).

#### EGFR

EGFR is one of the most frequently overexpressed proteins in NSCLC, found in both wild-type and mutated variants, with prevalence rates of over 11% in Caucasian and about 50% in Asian populations. It is also more common in non-smokers than smokers (Dogan et al. 2012; Rude Voldborg et al. 1997). About 66% of patients with *EGFR* mutation have the in-frame deletion in exon 19, while less than 30% have a point mutation in L858R exon 21. Rare mutations make up the remainder (Castellanos et al. 2017).

Currently, there are several options for first-line treatment in advanced NSCLC, but different variants of mutations respond differently to the administered drugs, which are TKIs. These drugs work by preventing the phosphorylation process of compounds in the EGFR pathway, thus limiting cell division, causing cell cycle arrest, and initiating apoptosis (Abdelgalil et al. 2020). The first- and second-generation drugs work as reversible inhibitors of ATP binding to the TK domain, while third-generation drugs bind irreversibly (Gelatti et al. 2019). The most common variant, exon 19 deletion, responds better to first-line treatment (such as *erlotinib* or *gefitinib*) than L858R. However, most EGFR-mutated cancers can develop resistance caused by a T790M mutation, which can be pre-existent or appear after implementing the TKIs (Castellanos et al. 2017). To address this resistance, a new approach has been taken that introduces an irreversibly binding drug called *osimertinib*. Initially, it was used as a second-line treatment in T790M-mutated NSCLC and significantly extended OS in patients. The genuine success of its implementation also transferred to first-line treatment. The FLAURA phase 3 trial compared *osimertinib* with first-generation TKIs, showing that the PFS was 18.9 and 10.2 months in *osimertinib* and standard TKIs (Soria et al. 2018). The findings imply that *osimertinib* treatment, irrespective of the presence of T790M mutation, improves overall survival (OS) and causes fewer adverse events (AEs) compared to standard treatment. Additionally, both approaches outperform platinum-based chemotherapy. However, the sequential approach gives more consistent results, with the *osimertinib* treatment implemented only after the gain of T790M mutation in *EGFR*-mutated NSCLC (Leonetti et al. 2019).

#### KRAS

*KRAS* mutation occurs in 27.5% of NSCLC, mostly in LUAD (Judd et al. 2021). The most common variants are G12C (40%), G12V (19%), and G12D (15%). It is more often detected in women than men (Judd et al. 2021). Another study shows a correlation between smoking and the presence of *KRAS* mutations in 34% of patients diagnosed with NSCLC (Dogan et al. 2012). The protein product of this mutation, the consistently active form of KRAS, influences several pathways, with the most significant impact on the MAPK/ERK pathway, which causes overstimulation of cell proliferation and growth. KRAS mutated protein is hard to target, as its binding is not as tight as expected (Salgia et al. 2021). A recent trial of a new drug, *sotorasib*, a G12C KRAS protein irreversible inhibitor, shows promising effects in treatment. With a 37.1% response rate and 80.6% disease control, the PFS of patients who used this drug is 6.8 months, and the OS is 12.5 months, with 19.8% of grade 3 AES and 0.8% of grade 4 events. This drug binds to GDP-KRAS, the inactive form of the KRAS protein, and prevents it from changing into the GTP-KRAS form, which causes oncogenesis when the G12C mutation is present. The drug use is limited and is usually implemented as second-line treatment in advanced malignant NSCLC. Although superior to platinum-based chemotherapy, the outcome of the treatment varies depending on other mutations and the tolerability of the drug itself (Skoulidis et al. 2021).

#### B-RAF

The *B-RAF* proto-oncogene is commonly mutated in 2–4% of NSCLC cases, with most of these mutations occurring in LUADs (> 85%) and in current smokers. Different variants of B-RAF mutations determine the differentiation, with two main types identified—the V600E variant accounting for approximately 50% of all B-RAF mutations and non-V600E variants comprising the remainder. It has been reported that wild-type B-RAF patients have more prolonged OS than *B-RAF* mutated patients in those with EGFR mutations (O’Leary et al. 2019; Baik et al. 2017). *B-RAF* encodes a serine/threonine kinase, part of the MAPK/ERK pathway. Currently, two targets for inhibiting this pathway are available—B-RAF inhibitors (B-RAFi) or MEK inhibitors (MEKi). These drugs were previously used in metastatic melanoma and are now being used in NSCLC. Monotherapy with B-RAF inhibitors is not sufficient. Thus, combination therapy using both types of drugs is necessary to enhance cell cycle arrest and activate apoptosis. In clinical trials, treatment with dabrafenib and trametinib resulted in a 63% response rate and approximately 9.7 months of progression-free survival. The most common AEs reported in various trials were pyrexia, ALT increase, and vomiting. These findings suggest that BRAFi and MEKi therapy is effective and provides a promising treatment option for patients with *B-RAF* mutation (Baik et al. 2017; Planchard et al. 2017).

### ALK/ROS1 gene rearrangement

The most common mutation of the *ALK* gene in NSCLC is the *ALK-R* gene rearrangement, which can be detected in up to 6% of patients. The *ALK* gene commonly fuses with *EML4*, *KIF5B*, *KLC1*, and *TRP* in cancers, with *EMLA4* being the most frequent partner gene in NSCLC. These fusions increase the expression of an ALK protein—TK (Holla 2017; Du et al. 2018; Camidge et al. 2019). The *ROS1* rearrangement is another common targetable mutation in NSCLC, present in 1–2% of patients. CD74-ROS1 and SLC34A2-ROS1 gene fusions are the most frequent variants, resulting in constant TK activity. Both fusions cause phosphorylation, which activates components in several pathways, such as MAPK/ERK, PI3K, and PLC-γ, leading to uncontrolled cell proliferation and inhibiting cell termination (Holla 2017; Mao and Wu 2017a, b). The proteins resulting from the *EML4-ALK* and both *ROS1* chromosomal rearrangements are target molecules for treating NSCLC *ALK/ROS1*.

The first drug approved for *ALK* or *ROS1* pathway signalling inhibition was *crizotinib*, which has an objective response rate (ORR) of 72% that is fast and durable. The median PFS was 19.3 months, and the median OS was 52.4 months when such mutations were present (Shaw et al. 2014; Shaw et al. 2019; Moro-Sibilot et al. 2019; Recondo et al. 2018). However, *crizotinib* lacks a durable response due to resistance gained toward it, resulting from the L1196M mutation. *Alectinib* is a drug introduced into clinical trials to respond to this mutation, with a 48–55% response rate in *ALK*-positive crizotinib-resistant NSCLCs. It has also been used in patients with no previous treatment, with a 93.5% ORR (Holla 2017). Another study showed that *alectinib* had significantly higher PFS (68.4% vs 48.7%) and ORR (82% vs 75.5%) over 12 months, in combination with lower toxicity (41% vs 50%) than *crizotinib*. These findings suggest that *alectinib* is solid choice to be used in first-line treatment for ALK-positive NSCLC patients (Camidge et al. 2019; McKeage 2015).

*Brigatinib* is a next-generation ALK inhibitor (since 2020 is approved by FDA as first-line treatment in NSCLC), and comparison with *crizotinib* favours the newer drug. The comparison shows a rate of PFS, 67% vs 43% (12-month period), a response rate of 71% vs 60%, and an intracranial response of 78% vs 29% (Camidge et al. 2018). Another study described a 24.0-month vs 11-month PFS in 24.9 months, with additional information about a delayed median time to worsening compared to *crizotinib* (Camidge et al. 2020).

### CRISPR and nanotechnology

In this section, clustered regularly interspaced short palindromic repeat (CRISPR) and nanoparticles as the latest approaches in NSCLC treatment will be discussed separately and cooperatively. CRISPR is a genome-editing tool helpful in developing personalised therapies, whereas nanotechnology is mainly used to improve drug delivery systems. The latest research shows promising results of combining nanomedicine and CRISPR to improve delivery and activation for in vivo genome editing therapy in NSCLC (Taha et al. 2022).

#### CRISPR-CAS9/12A

CRISPR was initially discovered as part of an adaptive defence system in the *Escherichia coli* genome and was later adapted into an in vivo editing and targeting tool (Wiedenheft et al. 2012). What creates CRISPR is a small guide RNA that forms a complex with the inactivated DNA-binding protein Cas9 and together they attach to a precise region of the genome. With the production of RNA-guided nucleases (RGNs) like Cas9, allowing to obtain tailored specificities, many targets in the genomes of cells can be reached. The first step in genome editing involves creating a double-stranded DNA break at specific loci using the CRISPR-Cas system. The resulting break can be repaired by different pathways, including nonhomologous end-joining (NHEJ) and homology-directed repair (HDR), with HDR being a more precise mechanism (Sander and Joung 2014). The CRISPR system is typically combined with Cas9 nuclease, which is derived from the bacterial CRISPR-associated protein nine nucleases from *Streptococcus pyogenes* (Sander and Joung 2014). However, other systems that use Cas12 nucleases have shown promising results in the treatment of NSCLC (Feng et al. 2022). Bijoya et al. suggests that CRISPR-Cas12a can be used as a multiplex genome-editing tool and a system to promote HDR instead of NHEJ, which can lead to more precise DNA targeting (Paul and Montoya 2020). In 2020, Sreedurgalakshmi et al. comprehensively described the use of CRISPR-Cas in NSCLC treatment, including several issues that may lead to treatment resistance, such as mutations, molecular rearrangement, and conformational changes in ATP-binding domains (Sreedurgalakshmi et al. 2021). Therefore, CRISPR holds promise in different strategies, such as drug target validation and screening, generating mutant cell models, and discovering new targets for chemotherapy.

Today, one of the most common approaches in cancer research is investigating the roles of specific enzymes and domains in tumour growth to find new therapeutic targets to support chemotherapy. CRISPR is efficient in overcoming acquired drug resistance, as indicated by mutations in *EGFR*, *KRAS*, *NRAS*, *B-RAF*, and other genes. Therefore, it is necessary to investigate mutations responsible for modulating the sensitivity of these targets (Tsukumo et al. 2020; Terai et al. 2021; Floc’h et al. 2018; Park et al. 2021; Raoof et al. 2019; Gammelgaard et al. 2019; Wang et al. 2016; Caiola et al. 2020; Guerra et al. 2020).

**Fig. 4:**
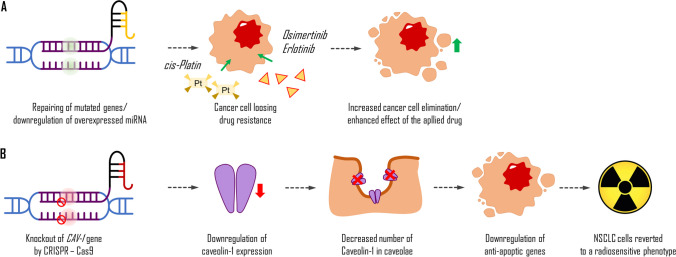
Potential application of Crisp-Cas system in the treatment of NSCLC. Increasing the possibilities of drugs used in the cancer treatment (**A**); caveolin-1 is a structural protein in caveolae. Its overexpression in cancer cells leads to the upregulation of anti-apoptotic genes. Thus, the knockout of the caveolin-1 (CAV-1) gene by CRISPR-Cas9-mediated gene editing leads to a decrease in its synthesis and a decrease in its number in caveolae. This process causes the downregulation of anti-apoptotic gene transcription in targeted NSCLC cells which reverts them to be sensitive to radiation (**B**)

Most patients diagnosed with advanced stages of NSCLC with *EGFR* mutations are treated with TKIs, but they often develop secondary resistance to drugs such as *dasatinib*, *erlotinib*, and *osimertinib*. Studies have shown the potential of the CRISPR system to overcome these issues or predict therapeutic outcomes by revealing genes and mediators, such as microRNA, responsible for drug sensitivity (Fig. 4A). For example, the efficacy of *dasatinib* may be evaluated by revealing molecular alterations of YES1 (Garmendia et al. 2019). *Erlotinib* resistance might be caused by the presence of microRNA-214 (miR-214) or depletion of insulin-like growth factor 1 receptor (Liao et al. 2017; Hussmann et al. 2017). Acquired resistance to *osimertinib* might be overcome with ERK inhibition (Li et al. 2020). Enforced expression of miR-204 can downregulate caveolin-1 (CAV-1) expression and resensitise NSCLC cells for *cisplatin*, another commonly used drug that interferes with DNA replication in fast-proliferating cells.

Moreover, the CRISPR-Cas system can also be used to improve more traditional treatment methods, such as radiotherapy, by targeting and suppressing factors responsible for radio-resistance, such as focal adhesion kinase in KRAS-mutant cells or gene knockout, which might revert NSCLC cells to a radiosensitive phenotype (Leiser et al. 2021; Moreno Roig et al. 2019; Tang et al. 2016). Recent research indicates that CAV-1 is highly expressed in some NSCLC cells, and the CRISPR-Cas9 knockout of that protein’s gene can revert such cells to a radiosensitive phenotype (Fig. 4B) (Leiser et al. 2021).

Overall, CRISPR has demonstrated great potential in different strategies such as drug target validation and screening, generating mutant cell models, discovering new targets for chemotherapy, and improving traditional treatment methods like radiotherapy. Further research using the CRISPR system could provide valuable insights into the mechanisms of cancer and help develop more effective therapies for NSCLC. The abovementioned studies were all pre-clinical and conducted mostly in vitro using human cell models or in vivo on mouse models incorporating xenografts. Such mouse xenograft models involve the transplantation of cells, e.g. modified by CRISPR-Cas or human tumour cells into immunodeficient or humanised mice.

Recent research has shown the potential of CRISPR in improving diagnostic tools for NSCLC, such as next-generation sequencing (NGS). Wang et al. have demonstrated the benefits of using the CRISPR-Cas9 system in the early detection of circulating tumour DNA in NSCLC (Wang et al. 2020b). This is crucial because late diagnosis is a significant challenge in treating this neoplasm. The ultimate treatment goal is to halt tumour metastasis, particularly to the brain, which is associated with a poor prognosis. The activated leukocyte cell adhesion molecule has been identified as a critical factor in brain metastasis formation, and its gene knockout using CRISPR has been suggested as a new therapeutic target (Justine et al. 2020). Other factors, such as myocardin, have been shown to promote pathways that enhance the epithelial-mesenchymal transition of NSCLC cells (Tong et al. 2020). Prior to the publication by Sreedurgalakshmi et al., only in vitro or in vivo studies using mouse models had been published. In 2020, the results of the first-in-human phase I clinical trial of CRISPR–Cas9 PD-1-edited T cells in patients with advanced NSCLC were reported (Lu et al. 2020). Twelve patients whose tumours were unresponsive to other available therapies were treated using this approach. The results showed that the treatment was generally safe and feasible, with a low frequency of off-target mutations (median 0.05%) (Lu et al. 2020).

To conclude, as indicated by the aforementioned reports, CRISPR-Cas9 technology holds great promise in the field of cancer research and treatment. It can contribute significantly to our understanding of cancer genomics by enabling the creation of genetically modified models of cancer within the laboratory setting. These models, in turn, aid researchers in comprehending the genetic mutations and molecular pathways underpinning various cancer types. This knowledge is pivotal in the development of targeted therapies. Furthermore, CRISPR-Cas9 can be employed to precisely target and edit the genes responsible for cancer initiation or progression. This capability has the potential to deactivate oncogenes or activate tumour suppressor genes, thus presenting an innovative approach to cancer treatment. Moreover, CRISPR technology can be effectively utilised to enhance immunotherapy by genetically modifying immune cells, such as T cells, to improve their efficiency in recognising and combating cancer cells. Another valuable application lies in addressing the significant challenge of drug resistance in cancer treatment. CRISPR technology plays a crucial role in elucidating how cancer cells develop resistance to chemotherapy or targeted therapies. This understanding can pave the way for the development of strategies to overcome drug resistance, ultimately enhancing the efficacy of cancer treatments. Additionally, this approach has the potential to usher in the era of personalised medicine. CRISPR-Cas9 could facilitate the development of personalised cancer treatments by analysing a patient’s tumour DNA and utilising CRISPR to target specific genetic mutations. This allows for the tailoring of treatments to align with an individual’s unique cancer profile. Lastly, in certain cases, genetic mutations directly contribute to cancer development. In such instances, CRISPR-Cas9 can be effectively applied in gene therapy approaches to correct these mutations or replace faulty genes, potentially leading to a genetic-level cure for cancer.

The convergence of cutting-edge technology and cancer research presents a promising future for more effective and personalised cancer treatments.

### Nanoparticles

Nanotechnology is rapidly becoming one of the most promising therapeutic strategies for treating widespread diseases such as diabetes, bacterial infections, cardiovascular diseases, and cancer (Mukherjee et al. 2019). Nanoparticles have been utilised for diagnosis and treatment, including active drug delivery and CRISPR-Cas systems (Mukherjee et al. 2019). Mukherjee et al. identified several nanomaterials, such as liposomal, polymeric, metal, and bio-nanoparticles, with promising theranostic effects in their 2019 study. These materials have been shown to improve drug delivery by increasing stability and site specificity, reducing the toxic effect on non-cancerous cells, and enabling a reduction in dosage frequency (Ngema et al. 2021). These benefits are significant for developing the CRISPR-Cas system for in vivo delivery and activation. When it comes to NSCLC treatment, albumin-based nanoparticles are currently being utilised across various stages of clinical trials to transport *paclitaxel* and *cisplatin.*

Three groups of carriers can be used for CRISPR-Cas delivery: biological, physical, and chemical. Among the chemical group, nanoparticles such as gold nanoparticles (AuNPs) and lipid nanoparticles (LNPs) have been demonstrated as a possible approach to the treatment of NSCLC (Taha et al. 2022). This strategy is expected to overcome the limitations of biological and physical methods, which can be immunogenic or toxic and mainly limited to ex vivo manipulations. AuNPs are bound with thiol-modified oligonucleotides, hybridised with a single-stranded donor oligonucleotide, complexed with Cas9 RNP, and coated with the endosomal disruptive polymer PAsp, which allows specific cell internalisation (Taha et al. 2022). However, AuNPs have a low level of HDR system induction.

Meanwhile, LNP technology is becoming increasingly popular due to its effectiveness in RNA-based vaccines against COVID-19. The method employs a combination of four types of lipids: cationizable (or ionisable) lipid, PEG lipid, helper phospholipid, and cholesterol. Its pH dependency and lack of proteins or peptides are thought to be responsible for its lower toxic or immunogenic level. Following the advancement of LNP technology, the first phase 1 clinical trial employing LNP-CRISPR took place in 2020 and a targeted organ was a human liver. Thus, such an approach holds promise for extending the scope of organs and genetic conditions amenable to targeting.

Given that most patients with NSCLC are diagnosed at advanced stages and develop secondary resistance to therapeutic methods, the development of both CRISPR and nanoparticle technology is essential for improving treatment outcomes.

## Conclusion

Conventional therapies, including surgery, radiotherapy, and chemotherapy, are still the primary treatments for NSCLC. However, despite significant development and improvement in these treatments, OS and PFS remain unsatisfactory, especially for more advanced stages of cancer. Therefore, developing new and more advanced therapies and studying them is crucial.

Emerging experimental methods in medicine, such as CRISPR and nanotechnology, are essential for developing highly personalised therapies. The CRISPR-Cas9 and Cas12a systems provide a variety of approaches in both therapeutic and diagnostic processes. It is worth mentioning that CRISPR can be used for drug target validation, generating mutant cell models, discovering new targets for chemotherapy, and developing screening tests. It is significant that CRISPR-Cas9 already shows promising results in in-human clinical trials using modified T cells of patients. Nanomedicine focuses on improving drug delivery systems, but its application extends to the precise transport of the abovementioned genome-editing tools. However, more comprehensive studies are required to evaluate the safety and efficacy of these techniques.

Immunotherapy has gained popularity over the years and has become one of the most promising approaches to NSCLC treatment. It provides an increase in PFS and OS while still being relatively safe for most patients. Its nature incorporates a patient’s immune system instead of relying on aggressive cytotoxic drugs, allowing combination with other primary NSCLC therapies, such as chemotherapy.

Conventional therapies are based on resecting a tumour or using cytotoxic agents. In contrast, modern methodology targets the cause of tumorigenesis, resulting in a lower impact on surrounding non-cancerous tissue. Current technology and its continuous improvement allow for a diverse approach to LC care. Combining well-established and innovative forms of treatment enables oncologists to exploit individual variables, such as mutations or genetic landscapes, and increase a patient’s chance of survival. Future research should focus on integrating these forms of treatment and taking advantage of knowledge about another molecular pathway for challenging of lung tumour development.

## Abbreviations

AE: Adverse effect
ALK: Anaplastic lymphoma kinase
AuNP: Gold nanoparticle
B-RAFi: B-RAF inhibitors
CRISPR: Clustered regularly interspaced short palindromic repeat
CTLA-4: Cytotoxic T-lymphocyte associated protein-4
EGFR: Epidermal growth factor receptor
HDR: Homology-directed repair
HIF: Hypoxia-induced factor
ICI: Immune checkpoint inhibitor
IGRT: Image-guided radiotherapy
KRAS: Kirsten rat sarcoma virus
LC: Lung cancer
LNP: Lipid nanoparticle
LUAD: Adenocarcinoma of the lung
mAb: Monoclonal antibody
MEKi: MEK inhibitors
NGS: Next-generation sequencing
NHEJ: Non-homologous end-joining
NSCLC: Non-small cell lung cancer/carcinoma
ORR: Objective response rate
OS: Overall survival
PD-1: Programmed death receptor-1
PD-L1: Programmed death ligand-1
PD-L2: Programmed death ligand-2
PFS: Progression-free survival
PS: Performance status
RATS: Robot-assisted thoracic surgery
RGRT: Respiratory-gated radiotherapy
SBRT: Stereotactic body radiation therapy
TK: Tyrosine kinase
TKI: Tyrosine kinase inhibitor
TSG: Tumor suppressor gene
VATS: Video-assisted thoracic surgery
VEGF: Vascular endothelial growth factor
VEGFR: Vascular endothelial growth factor receptor
